# Causal Relationship Between Mitochondrial Characteristics and Inflammatory Bowel Disease and Its Subtypes: Evidence for Mendelian Randomization

**DOI:** 10.1002/jgh3.70257

**Published:** 2025-08-19

**Authors:** Yichen Cai, Hancheng Fan, Jianmin Zhao, Guoxiang Fu

**Affiliations:** ^1^ Department of Pathology at Sir Run‐Run Shaw Hospital Zhejiang University School of Medicine Hangzhou China

**Keywords:** genome‐wide association studies, inflammatory bowel disease, Mendelian randomization, mitochondria

## Abstract

**Objective:**

The research was designed to explore the causal relationship between the changing landscape of 63 mitochondrial proteins and inflammatory bowel disease (IBD) by Mendelian randomization.

**Methods:**

Use SNPs significantly associated with IBD and their subtypes as instrumental variables. Various methods were utilized to assess the causal relation between mitochondrial function and the onset of IBD and their subtypes.

**Results:**

The results after FDR on *p* values indicated that Peptide chain release factor 1‐like was a causative factor for IBD. NADH dehydrogenase [ubiquinone] flavoprotein 2, hydroxymethylglutaryl‐CoA synthase 9, pyruvate carboxylase, apoptosis‐inducing factor 1, and transmembrane protein 70 with inflammatory bowel disease were identified as protective factors. Peptide chain release factor 1‐like and NADH dehydrogenase [ubiquinone] flavoprotein 2 were identified as causal and protective factors for IBD, respectively. In addition, GrpE protein homolog 1, 39S ribosomal protein L33, and tRNA pseudouridine synthase A are pathogenic in Crohn's disease, and Hydroxymethylglutaryl‐CoA synthase and Pyruvate carboxylase are protective factors. The MR‐presso method rejected outlier SNPs. The relative stability of the outcome data was assessed and validated at multiple levels by various statistical tests including pleiotropy test, heterogeneity test, and Steiger test.

**Conclusion:**

This study established a causal relationship between mitochondrial biological function and IBD, including its subtypes.

## 1 Introduction

Inflammatory bowel disease (IBD) represents the complex, multi‐factorial immunologically mediated inflammation of the gastrointestinal tract, typically chronic and prone to relapse [1]. As its etiology and pathogenesis have not been fully clarified, the clinical typology of IBD is mainly based on the site of occurrence as well as the clinical manifestations, of which the prevalent types are Crohn's disease (CD) and ulcerative colitis (UC) [2]. The CD presents discontinuous transmural inflammation which may spread throughout the gastrointestinal tract, characterized by distinctive histological granulomas. UC, on the other hand, exhibits inflammation localized to the mucosa, often involving the rectum and continuing to extend into the proximal part of the colon [3]. IBD is recognized as a worldwide health concern, experiencing an escalating prevalence in emerging urbanized nations and a stable, but still escalating, increase over westernized nations. Available clinical data show that the maximum incidence of the disease is mainly between the ages of 15 to 30, and that about 10% of cases are identified prior to adulthood [4, 5]. Current treatment modalities encompass biologics, immunomodulators, aminosalicylates, and corticosteroids [6, 7]. However, treatment outcomes vary significantly due to the heterogeneity of the disease among patients, posing a challenge to therapeutic strategies [8]. Therefore, it is of vital importance to further study the etiology and regulatory processes in IBD to identify novel treatment goals and develop useful intervention strategies. It is worth noting that distinctions among IBD subtypes may also result in variations in potential therapeutic targets.

Mitochondria, as unique double‐membrane organelles containing soluble substrates and distinct gene sets, are the major location where adenosine triphosphate (ATP) is synthesized [9]. In contrast, about 90% of ATP, which is the main energetic flux for cellular physiological and biochemical processes in the human body, is produced by oxidative phosphorylation in the mitochondria. This suggests the importance of mitochondrial structure and function in energy metabolism. Meanwhile, recent studies have shown that mitochondrial function in the gastrointestinal epithelium is important for sustaining intestinal health, and that there is a strong link between mitochondrial dysfunction and IBD [10, 11]. It is well known that significant shedding of intestinal epithelial cells occurs in IBD. This poses a higher challenge to the renewal function of intestinal stem cells [12]. Whereas mitochondrial respiration and metabolism conversion are critical drivers of the fate of Lgr5+ crypt base columnar stem cells, which are rich in leucine‐rich repeat‐containing G protein‐coupled receptor 5 (Lgr5^+^) [13, 14]. Paneth cells, which are closely related to intestinal immunity, have also been reported to be highly susceptible to CD‐related mitochondrial dysfunction [15]. In addition, the release of mitochondrial reactive oxygen species (ROS) and mitochondrial damage‐associated molecular patterns contribute to UC inflammation [16]. Various quality control mechanisms maintain mitochondrial pool health, with mitochondrial autophagy proven to prevent UC [17]. Evidence from these studies suggests that we IBD and development are closely related to changes in mitochondrial function. Targeting the underlying mitochondrial dysfunction may be a novel strategy for treating IBD. Thus, identifying mitochondrial targets that are significantly causally linked to IBD has far‐reaching therapeutic value.

In traditional epidemiological research, associations between exposure and outcomes may be influenced by unmeasured confounders and reverse causation, limiting the ability to draw causal inferences. Mendelian randomization (MR) studies utilize genome‐wide association study (GWAS) data, employing genetic variants as instrumental variables (IVs) [18]. These models aim to assess the associations between risk factors (exposure) and various outcomes (disease types), mitigating the impact of confounders and reversing the direction of causal inference [19]. This study aims to examine the causal relationship between mitochondrial proteins and inflammatory bowel disease through MR analysis based on GWAS data.

## 2 Materials and Methods

### 2.1 Data Selection

In this study, Mitochondrial GWAS data for 63 mitochondrial proteins were utilized as exposure variables. SNPs significantly linked to IBD and its subtypes (UC and CD) were chosen as IVs, with IBD‐related data as outcome variables [20].

### 2.2 Data Sources

GWAS data related to 63 kinds of mitochondrial proteins were obtained from databases such as UKB. Sixty‐three kinds of mitochondrial proteins include four kinds of GWAS data (Table S1) [21, 22, 23, 24]. GWAS summarized statistics about IBD were obtained from FinnGen + pan‐UKBB meta‐analysis results, which is the largest IBD GWAS data available (all variants recognized: 20 175 454 variants; total sample size: 377 277). Notably, the GWAS data associated with IBD includes statistics for UC and CD (Table 1) [25].

**TABLE 1:** GWAS data related to IBD.

ID	Name	Populations	Cases	Controls	All
K11_IBD_STRICT	Inflammatory bowel disease, strict (require KELA)	European	7625	369 652	377 277
K11_UC_STRICT2	Ulcerative colitis (strict definition, require KELA, min 2 HDR)	European	5034	371 530	376 564
K11_CD_STRICT2	Crohn disease (strict definition, require KELA, min 2 HDR)	European	1665	375 445	377 110

### 2.3 Correlation Analysis

**FIGURE 1:**
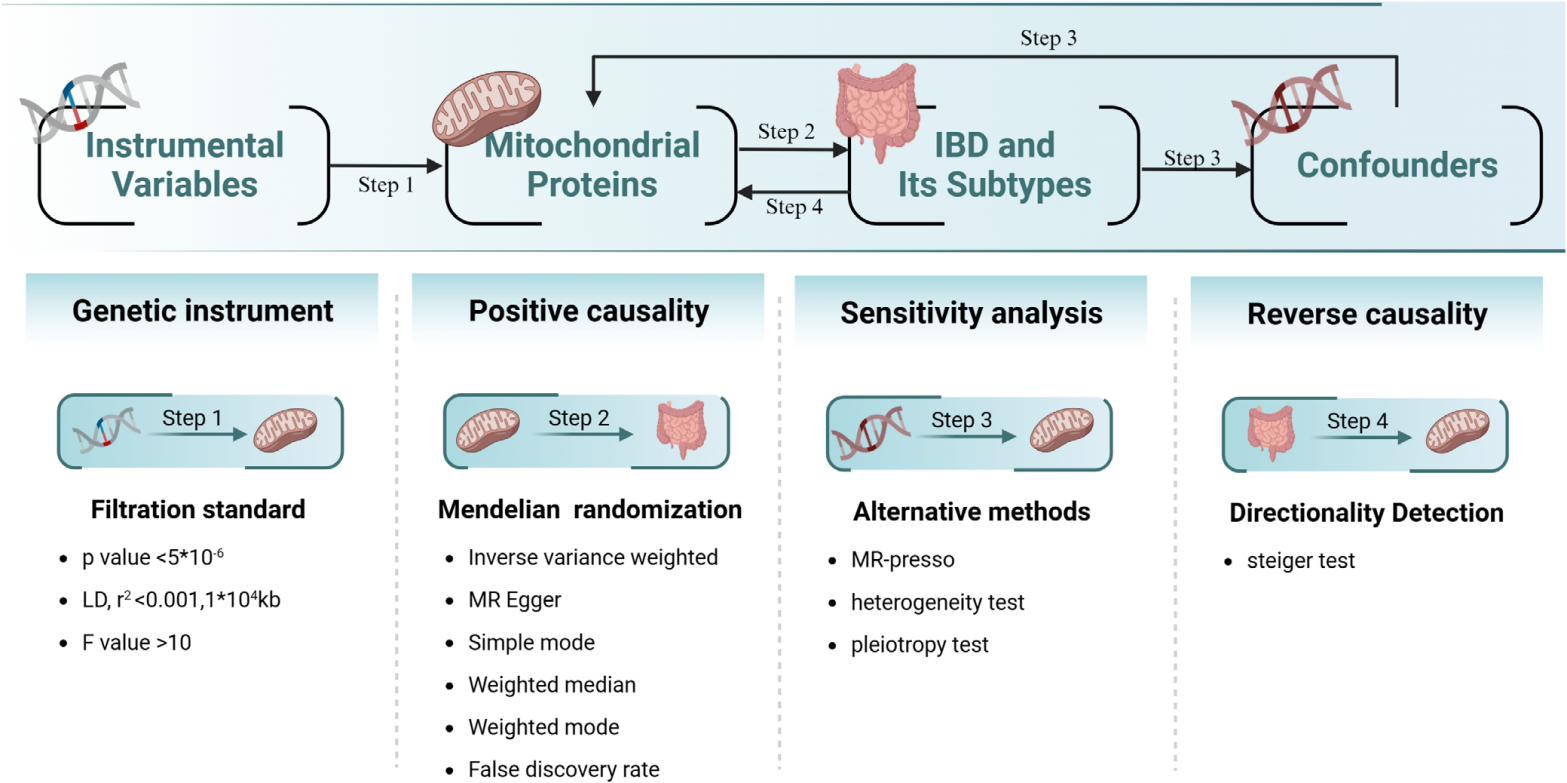
The methods and experimental design of Mendelian randomization.

MR analyses as a novel statistical method rely on three basic assumptions: (1) the assumption of correlation: the strong correlation is assumed to exist between IVs and exposure factor; (2) independence assumption: the IVs and the outcome variable, that is, IBD and its subtypes, are independent of any other confounders; (3) exclusivity assumption: the IVs can only influence IBD through regulation of mitochondrial activity, without involving any other pathway (Figure 1). In order to determine appropriate IVs, we initially chose SNPs that strongly related to IVs as IVs, with a selection criterion of *p* < 5e−08. However, when a *p* value of 5e−08 was chosen, the number of available SNPswas too limited, with only 10 mitochondrial proteins having multiple SNPs; so we finally chose *p* < 5e−06 as the criterion [26] (Table S2).

### 2.4 Linkage Disequilibrium

Linkage disequilibrium (LD) is the chance that alleles belonging to two or more gene loci will occur together on a chromosome more often than randomly. The selection criteria are the following: (1) kb > 10 000; (2) *r* ^2^ < 0.001. And “kb” represents the range of the regional LD, while “*r* ^2^” is evaluated from 0 to 1. The closer the *r* ^2^ value is to 0, the more likely it is to be perfectly linked and balanced; the more likely the assignment of two SNPs is to be completely random [27].

### 2.5 Elimination of Weak IVs

MAF represents minor allele frequency, and SNPs refer to mutations that occur in a certain proportion of the population. Typically, when performing SNP filtering, the MAF threshold should be greater than 0.01, meaning that the mutation exists in at least 1% of the population [28].

The *F* statistic is used to evaluate the bias arising from weak instrumental variables for IVs. This paper specifies that it will classify SNPs as weak IVs when *F* < 10 and non‐weak IVs when *F* > 10. *F* values are given by the following formula:

$$F=\frac{N-k-1}{k}\times \frac{R^{2}}{1-R^{2}}$$

where *N* expresses the sample size of the exposure variable, *K* expresses the number of IVs, and *R* ^2^ indicates the proportion of IVs that accounted for the exposure. *R* ^2^ is given by the formula shown below:

$$R^{2}=\left(1-\mathrm{MAF}\right)\times \mathrm{MAF}\times \beta^{2}$$

*β* represents the SNPs' influence size on the exposure factor.

### 2.6 MR Analysis

Five approaches (Inverse Variance Weighting (IVW), MR Egger, Weighted Median, Simple Mode and Weighted Mode) have been used in this study to ascertain causality between mitochondrial proteins (exposure factors) and IBD (outcome factors) (Tables S3–S5). Of these, the IVW method was considered to be the primary measure of the reliability of MR analyses with multiple SNPs; when *p* < 0.05, it indicates that the causality was judged as a positive result. Moreover, the “TwoSampleMR” R package is used to visualize the outcome, including scatter and forest plots, plus sensitivity analysis maps [29].

### 2.7 Horizontal Pleiotropy Test

The principle of MR does not allow for IVs to directly influence the results without the exposure factor, which would lead to horizontal pleiotropy in the test results. Therefore, we utilized the MR Polytropic Residuals and Outliers (MR‐PRESSO) testing to examine SNPs that brought about horizontal polytropy (Nb Distribution = 1000, Significant Threshold = 0.05), which was performed by the “MR‐PRESSO” software R package. MR‐PRESSO is determined by calculating the IVW result with the removal of the SNP and the residual sum of squares of the effect and IVW result of that SNP to the line of fit calculated with the removal of that SNP, and the sum of squares of the residuals from the IVW result, which ultimately yields the distance from that SNP to the line of fit calculated by removing that SNP. The greater the value of the sum of squared residuals (distance) calculated for each SNP, the more significant the horizontal pleiotropy was indicated. SNPs with horizontal pleiotropy will be removed [30].

### 2.8 Sensitivity Analysis

Because of variations of the platform, experiments, and populations in GWAS data, instrumental variables may exhibit heterogeneity; thereby affecting the results of MR. This study utilized IVW and MR‐Egger measures for evaluating the heterogeneity of MR, with *p* < 0.05 indicating the existence of MR heterogeneity.

In addition, if an instrumental variable affects the results via factors outside of the exposure variable, it is indicative of pleiotropy. The presence of polymorphism would break the assumptions of independence and exclusivity. For assessing multiplicity and the robustness of the study results, we conducted the MR‐Egger intercept testing. A *p* value of less than 0.05 indicates that there is pleiotropy within the samples.

The “leave‐one‐out” method was used to perform sensitivity analysis. After systematically eliminating results associated with individual SNPs, they were assessed for being anomalous outliers based on a forest plot, which was also used to observe the stability of the results after the elimination of each SNP. The R programming language for generating funnel plots was also used for the sensitivity assessment, and the symmetry that the SNPs presented in the funnel plots was used to assess the reliability of the results [31].

### 2.9 Steiger Test

As a tool used to examine causality between genotype and phenotype in genetics, directional testing is necessary for MR [32]. The Steiger test is a common statistical measure for testing the directionality of correlation coefficients. Its principle is based on conducting a one‐tailed test on the correlation coefficient to determine whether the relationship between two variables is positive or negative. This testing method can help researchers determine whether genotype has a significant impact on phenotype, as well as the direction of this impact.

### 2.10 False Discovery Rate Control

The false discovery rate (FDR) is the proportion of false positives (rejection of the null hypothesis H0 when it is true) among all significant test results. The classical Benjamini–Hochberg (BH) method is a technique used to control the FDR, ensuring that FDR ≤ *p*. The BH method adjusts the original hypotheses to ensure the maximization of true positive discoveries while controlling the FDR. In the BH method, if there are N hypothesis tests, the *p* value for each test is calculated. The calculated *p* values are then ranked as an increasing sequence, and starting from the smallest *p* value, they are compared according to the following formula, where K is the ranking. The largest *p* value satisfying the inequality can then be found at the K position, and it can be considered significant, while the remaining *p* values are not significant [33].

$$p\left(K\right)\leq p\times \frac{K}{N}$$

### 2.11 Statistical Analyses

We used the R software (version 4.3.2) to assess causality in MR analyses. The threshold of statistical significance was *p* values less than 0.05.

## 3 Results

### 3.1 Dataset Screening

**FIGURE 2:**
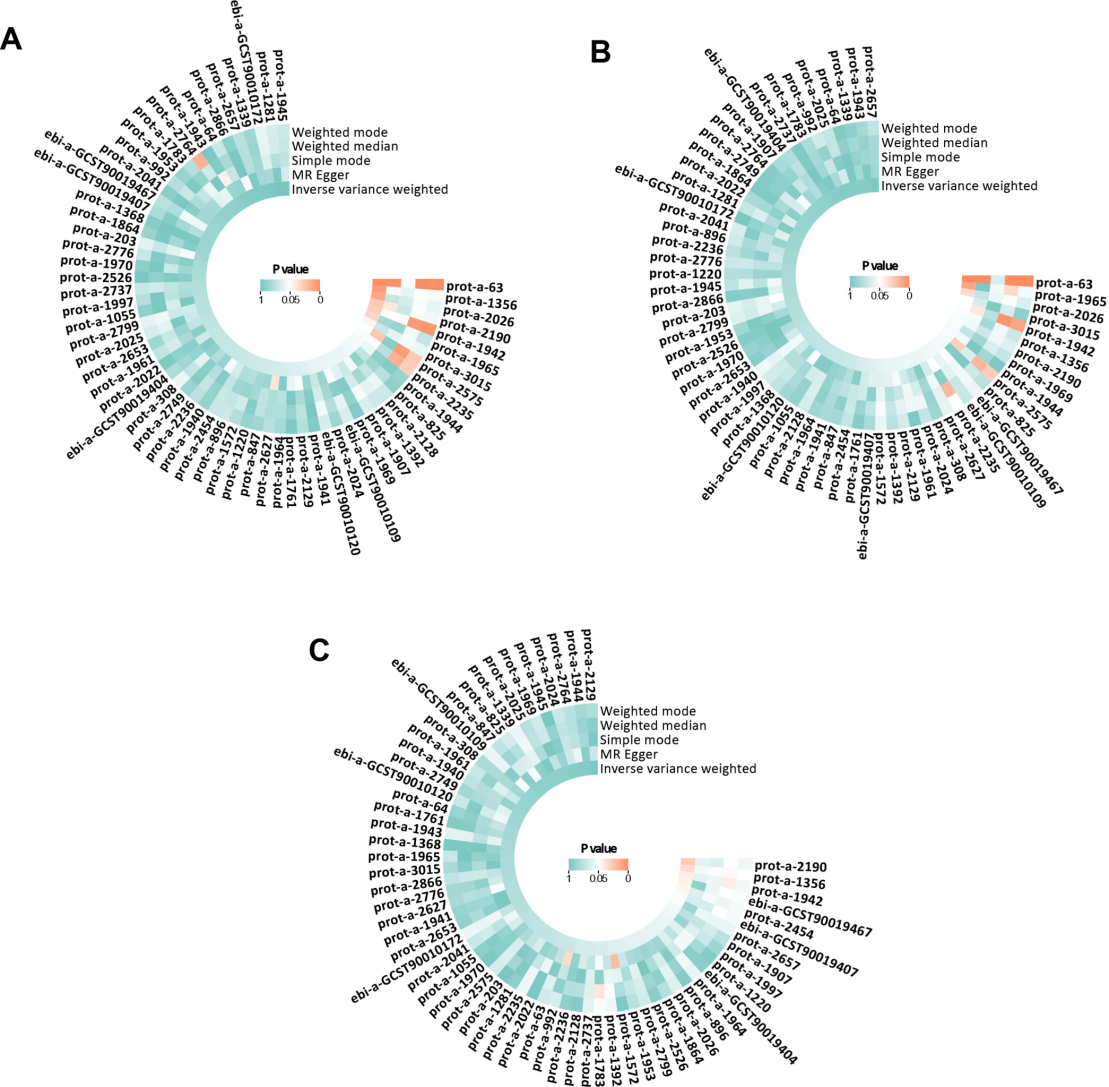
Five MR methods screened for significant mitochondrial proteins associated with IBD and its subtypes.

Nine proteins with *p* < 0.05 for IVW were tentatively considered to be causally associated with IBD: prot‐a‐63 (apoptosis‐inducing factor 1, mitochondria), prot‐a‐1356 (hydroxymethylglutaryl‐CoA synthetase, mitochondria), prot‐a‐2026 (NADH dehydrogenase [ubiquinone] flavoprotein 2 (NDUFV2), mitochondria), prot‐a‐2190 (pyruvate carboxylase, mitochondria), prot‐a‐1942 (39S ribosomal protein L33, mitochondria), prot‐a‐1965 (peptide chain release factor 1‐like, mitochondria), prot‐a‐3015 (transmembrane protein 70, mitochondria). Among them, prot‐a‐63 (apoptosis‐inducing factor 1, mitochondria), prot‐a‐1965 (peptide chain release factor 1‐like, mitochondria) and prot‐a‐2026 (NDUFV2, mitochondria) were significantly IVW in UC, prot‐a‐2190 (Pyruvate carboxylase, mitochondrial), prot‐a‐1356 (Hydroxymethylglutaryl‐CoA synthase, mitochondrial) with prot‐a‐1942 (39S ribosomal protein L33, mitochondria) in the CD was significantly IVW. Notably, prot‐a‐1942 (39S ribosomal protein L33, mitochondria) in the CD was significantly IVW with ebi‐a‐GCST90019467 (GrpE protein homolog 1, mitochondrial measurement) also had significant *p* values for IVW, which was not found in IBD. In the end, we initially screened seven mitochondrial proteins that were significantly associated with IBD, three mitochondrial proteins that were significantly associated with UC, and five mitochondrial proteins that were significantly associated with CD (Figure 2 and Tables S6–S8). The GWAS‐ID, sample size, and number of SNPs for mitochondrial proteins with positive results are given in the table below (Table 2).

**TABLE 2:** Mitochondria‐related GWAS data.

Gwas‐id	Sample size	SNPs	Exposure	Populations	IBD	UC	CD
prot‐a‐63	3301	10 534 735	Apoptosis‐inducing factor 1, mitochondria	European	+	+	
prot‐a‐1356	3301	10 534 735	Hydroxymethylglutaryl‐CoA synthetase, mitochondria	European	+		+
prot‐a‐2026	3301	10 534 735	NADH dehydrogenase [ubiquinone] flavoprotein 2, mitochondria	European	+	+	
prot‐a‐2190	3301	10 534 735	Pyruvate carboxylase, mitochondria	European	+		+
prot‐a‐1942	3301	10 534 735	39S ribosomal protein L33, mitochondria	European	+		+
prot‐a‐1965	3301	10 534 735	Peptide chain release factor 1‐like, mitochondria	European	+	+	
prot‐a‐3015	3301	10 534 735	Transmembrane protein 70, mitochondria	European	+		
prot‐a‐2454	3301	10 534 735	tRNA pseudouridine synthase A, mitochondrial	European			+
ebi‐a‐GCST90019467	10 708	15 566 219	GrpE protein homolog 1, mitochondrial measurement	European			+

### 3.2 SNP Screen

Horizontal pleiotropy of SNPs was examined with the R‐programmed MR‐PRESSO package. In the MR analysis with IBD shown to be result, rs28929474 and rs78745329 were excluded, which were from prot‐a‐1942, and prot‐a‐63. rs78745329 was excluded in the MR analysis with UC as the outcome, which was derived from prot‐a‐63. rs12566888, rs35121814, rs144487948 were excluded in the MR analysis with CD as the outcome, and the first two SNPs were derived from ebi‐a‐GCST90019467 and the latter one from prot‐a‐2190. The number of SNPs used between each exposure and outcome is given in the following table (Table 3).

**TABLE 3:** Mitochondrial dataset tool variation scale.

Exposure ID	Outcome	SNPs
prot‐a‐1356	Inflammatory bowel disease	11
prot‐a‐1942	Inflammatory bowel disease	10
prot‐a‐1965	Inflammatory bowel disease	10
prot‐a‐2026	Inflammatory bowel disease	7
prot‐a‐2190	Inflammatory bowel disease	11
prot‐a‐3015	Inflammatory bowel disease	9
prot‐a‐63	Inflammatory bowel disease	12
prot‐a‐1965	Ulcerative colitis	10
prot‐a‐2026	Ulcerative colitis	6
prot‐a‐63	Ulcerative colitis	12
ebi‐a‐GCST90019467	Crohn's disease	9
prot‐a‐1356	Crohn's disease	11
prot‐a‐1942	Crohn's disease	11
prot‐a‐2190	Crohn's disease	10
prot‐a‐2454	Crohn's disease	9

### 3.3 Sensitivity Analysis

**FIGURE 3:**
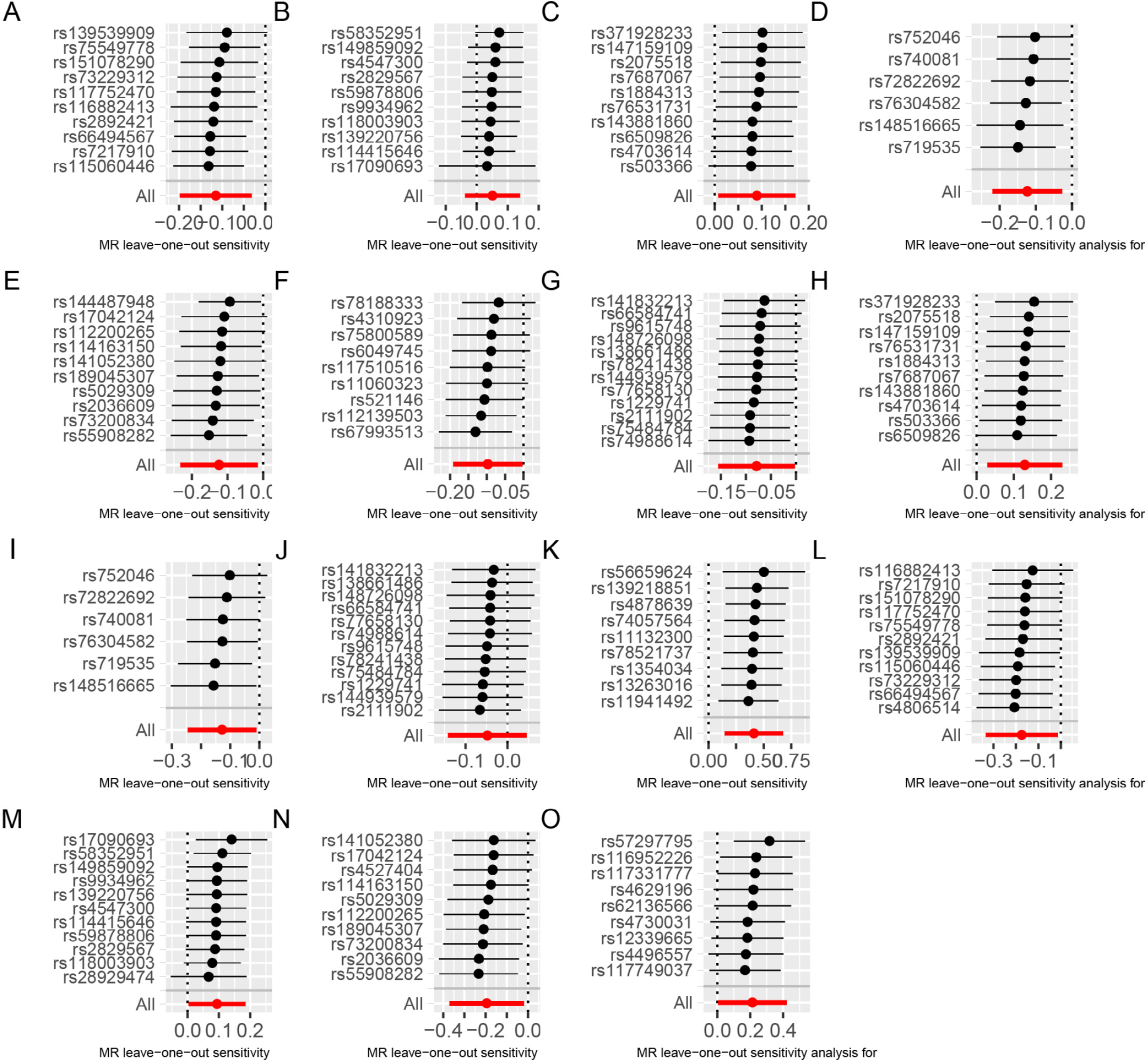
MR leave‐one‐out sensitivity analysis for exposure on outcome. (A) leave‐one‐out analysis for “hydroxymethylglutaryl‐CoA synthase, mitochondrial” on “inflammatory bowel disease”; (B) leave‐one‐out analysis for “39S ribosomal protein L33, mitochondrial” on “inflammatory bowel disease”; (C) leave‐one‐out analysis for “peptide chain release factor 1‐like, mitochondrial” on “Inflammatory bowel disease”; (D) leave‐one‐out analysis for “NADH dehydrogenase [ubiquinone] flavoprotein 2, mitochondrial” on “inflammatory bowel disease”; (E) leave‐one‐out analysis for “pyruvate carboxylase, mitochondrial” on “Inflammatory bowel disease”; (F) leave‐one‐out analysis for “transmembrane protein 70, mitochondrial” on “inflammatory bowel disease”; (G) leave‐one‐out analysis for “apoptosis‐inducing factor 1, mitochondrial” on “inflammatory bowel disease”; (H) leave‐one‐out analysis for “peptide chain release factor 1‐like, mitochondrial” on “ulcerative colitis”; (I) leave‐one‐out analysis for “NADH dehydrogenase [ubiquinone] flavoprotein 2, mitochondrial” on “Ulcerative colitis”; (J) leave‐one‐out analysis for “apoptosis−inducing factor 1, mitochondrial’ on “ulcerative colitis”; (K) leave‐one‐out sensitivity analysis for “GrpE protein homolog 1, mitochondrial measurement” on “Crohn disease”; (L) leave‐one‐out analysis for “hydroxymethylglutaryl‐CoA synthase, mitochondrial” on “Crohn disease”; (M) leave‐one‐out analysis for “39S ribosomal protein L33, mitochondrial” on “Crohn disease”; (N) leave‐one‐out sensitivity analysis for “pyruvate carboxylase, mitochondrial” on “Crohn disease”; (O) leave‐one‐out analysis for “tRNA pseudouridine synthase A, mitochondrial” on “Crohn disease.”

**FIGURE 4:**
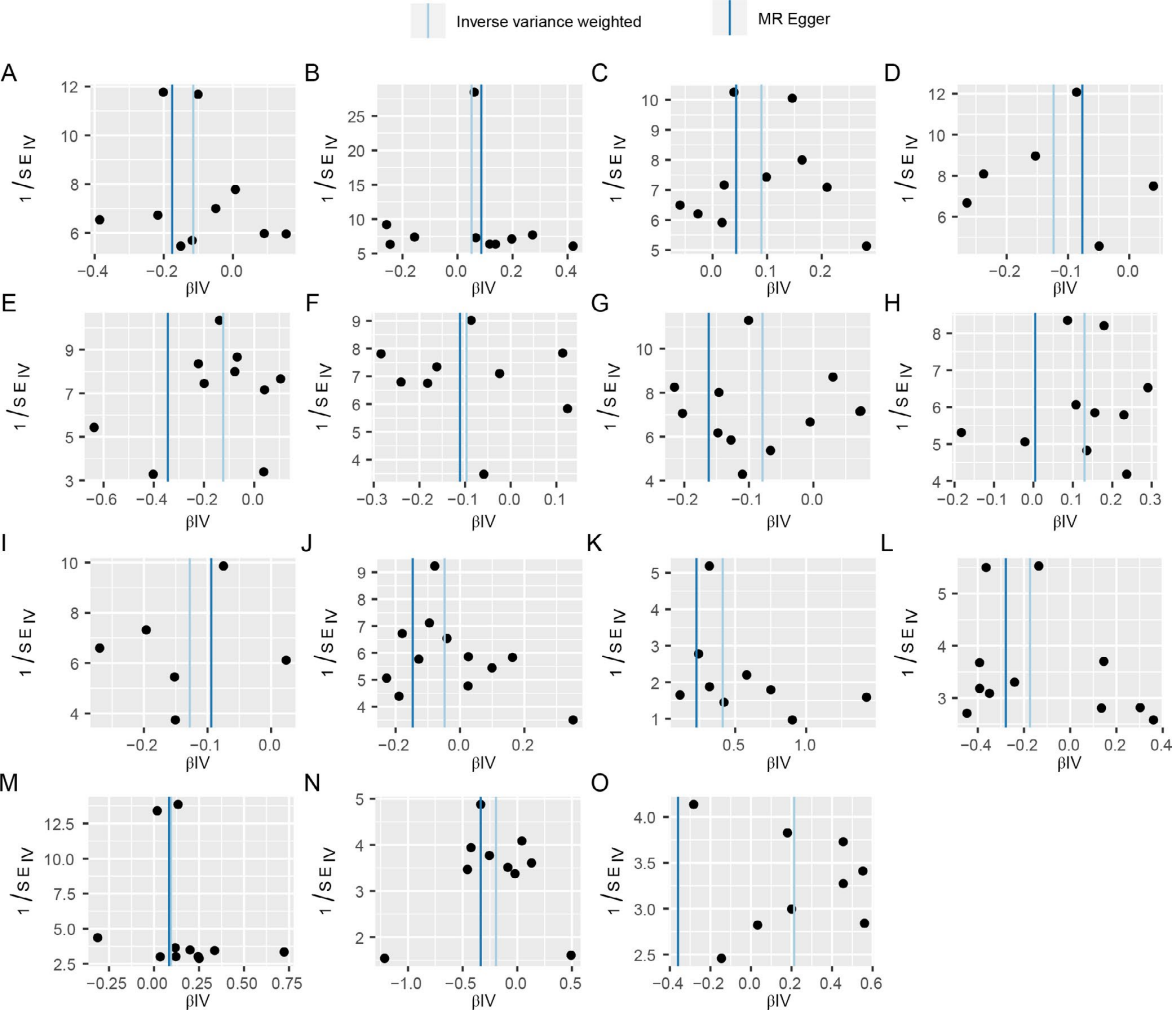
Mendelian randomization funnel plot for exposure on outcome. (A) MR funnel plot for “hydroxymethylglutaryl‐CoA synthase, mitochondrial” on “inflammatory bowel disease”; (B) Mendelian randomization funnel plot for “39S ribosomal protein L33, mitochondrial” on “inflammatory bowel disease”; (C) Mendelian randomization funnel plot for “peptide chain release factor 1‐like, mitochondrial” on “inflammatory bowel disease”; (D) Mendelian randomization funnel plot for “NADH dehydrogenase [ubiquinone] flavoprotein 2, mitochondrial” on “inflammatory bowel disease”; (E) mendelian randomization funnel plot for “pyruvate carboxylase, mitochondrial” on “inflammatory bowel disease”; (F) Mendelian randomization funnel plot for “transmembrane protein 70, mitochondrial” on “inflammatory bowel disease”; (G) Mendelian randomization funnel plot for “apoptosis‐inducing factor 1, mitochondrial” on “Inflammatory bowel disease”; (H) Mendelian randomization funnel plot for “peptide chain release factor 1‐like, mitochondrial” on “ulcerative colitis”; (I) Mendelian randomization funnel plot for “NADH dehydrogenase [ubiquinone] flavoprotein 2, mitochondrial” on “ulcerative colitis”; (J) Mendelian randomization funnel plot for “apoptosis‐inducing factor 1, mitochondrial” on “ulcerative colitis”; (K) Mendelian randomization funnel plot for “GrpE protein homolog 1, mitochondrial measurement” on “Crohn disease”; (L) mendelian randomization funnel plot for “hydroxymethylglutaryl‐CoA synthase, mitochondrial” on “Crohn disease”; (M) Mendelian randomization funnel plot for “39S ribosomal protein L33, mitochondrial” on “Crohn disease”; (N) Mendelian randomization funnel plot for “pyruvate carboxylase, mitochondrial” on “Crohn disease”; (O) Mendelian randomization funnel plot for “tRNA pseudouridine synthase A, mitochondrial” on “Crohn disease.”

Heterogeneity and pleiotropy were not present in MR analysis with IBD as the outcome, except for prot‐a‐1942, where heterogeneity was present. In MR analysis with UC as the outcome, no multivariate heterogeneity existed. No SNPs were considered to be present with pleiotropy in the MR analysis with CD as the outcome. Regarding this situation of heterogeneity with no pleiotropy in prot‐a‐1942, it is usually assessed using the Weighted Median method or the IVW‐multiplicative random effects model. The results of the Weighted Median method in prot‐a‐1942 remained significant (*p* < 0.05); meanwhile, in order to avoid the influence caused by heterogeneity, this paper then adopts the following MR analysis using the IVW‐multiplicative random effects model to conduct. Moreover, this paper took to verify the directionality of MR analysis using Steiger's directionality test, and all the results supported the causal relationship of mitochondrial proteins affecting IBD and its subtypes (Tables 4 and S9–S18). The Mendelian randomization leave‐one‐out sensitivity analysis shows no significant change in causality after removing a particular locus, and the effect remains stable, indicating that the model is relatively robust (Figure 3). The funnel plot shows a symmetrical shape, indicating that the distribution of the different genotypes or phenotypes in the experiment is even, in line with Mendel's prediction (Figure 4).

**TABLE 4:** Heterogeneity tests, pleiotropy test, and Steiger test *p* values for IBD and its subtypes.

Disease	GWAS‐ID	Heterogeneity tests		Pleiotropy test	Steiger test
		MR Egger	IVW		
Inflammatory bowel disease	prot‐a‐1356	0.324432	0.358268	0.461571	1.2266E−55
	prot‐a‐1942	0.003698	0.005033	0.606033	2.757E−146
	prot‐a‐1965	0.82928	0.873371	0.644476	1.289E−58
	prot‐a‐2026	0.507427	0.605262	0.606894	5.673E−36
	prot‐a‐2190	0.943378	0.588231	0.077953	1.4422E−57
	prot‐a‐3015	0.305231	0.401514	0.915457	4.7369E−44
	prot‐a‐63	0.897198	0.677512	0.091416	3.4741E−60
Ulcerative colitis	prot‐a‐1965	0.8391081	0.806857	0.3226653	2.3507E−58
	prot‐a‐2026	0.7040396	0.8088294	0.756921	2.1187E−36
	prot‐a‐63	0.8495803	0.8120621	0.2874596	1.8846E−60
Crohn disease	ebi‐a‐GCST90019467	0.84617504	0.850168019	0.436474664	4.9597E−74
	prot‐a‐1356	0.4867501	0.532200383	0.488887368	5.37E−56
	prot‐a‐1942	0.33865693	0.422834617	0.829172489	5.28E−292
	prot‐a‐2190	0.40986916	0.471329499	0.554248333	2.3912E−53
	prot‐a‐2454	0.59423492	0.34199229	0.105089832	1.2979E−46

### 3.4 Mendelian Randomization

In the MR analysis with IBD as the outcome, prot‐a‐1942 was no longer significant because it was tested using Inverse variance weighted (multiplicative random effects). In the MR analysis with UC as the outcome, this paper utilized the MR‐PRESSO method to exclude the outlier SNP: rs78745329 in prot‐a‐63, which resulted in the IVW result between prot‐a‐63 and UC no longer being significant. Therefore, we visualized the IVW results for the remaining 13 MRs and took the FDR approach to correct the *p* value (Table S19).

**FIGURE 5:**
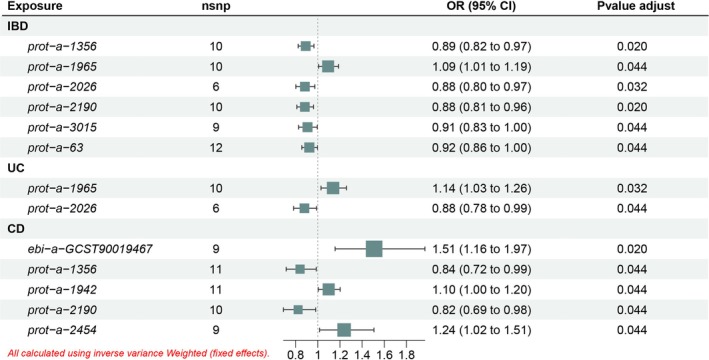
Causality between mitochondrial proteins and IBD and its subtypes. Abbreviations: CD, Crohn's disease; IBD, inflammatory bowel disease; UC, ulcerative colitis.

The final results showed that in IBD, with the exception of prot‐a‐1965 (peptide chain release factor 1‐like) as a risk factor (OR = 1.09; 95% CI = 1.01–1.19), prot‐a‐1356 (hydroxymethylglutaryl‐CoA synthase) (OR = 0.89; 95% CI = 0.82–0.97), prot‐a‐2026 (NDUFV2) (OR = 0.88; 95% CI = 0.80–0.97), prot‐a‐2190 (pyruvate carboxylase) (OR = 0.88; 95% CI = 0.81–0.96), prot‐a‐3015 (transmembrane protein 70) (OR = 0.91; 95% CI = 0.83–0.96), prot‐a‐63 (apoptosis‐ inducing factor 1) (OR = 0.92; 95% CI = 0.86–1.00) were protective factors. In UC, prot‐a‐1965 (peptide chain release factor 1‐like) (OR = 1.14; 95% CI = 1.03–1.26) was a risk factor and prot‐a‐2026 (NDUFV2) (OR = 0.88; 95% CI = 0.78–0.99) as a protective factor, both in the same direction as in IBD. In CD, ebi‐a‐GCST90019467 (GrpE protein homolog 1) (OR = 1.51; 95% CI = 1.16–1.97), prot‐a‐1942 (pyruvate carboxylase) (OR = 1.10; 95% CI = 1.00–1.20) were associated with prot‐a‐2454 (hydroxymethylglutaryl‐CoA synthase) (OR = 1.24; 95% CI = 1.02–1.51) as a risk factor and prot‐a‐1356 (39S ribosomal protein L33) (OR = 0.84; 95% CI = 0.72–0.99), and prot‐a‐2190 (tRNA pseudouridine synthase A) as a defense elements. Notably, ebi‐a‐GCST90019467 (GrpE protein homolog 1) and prot‐a‐1942 (pyruvate carboxylase) did not show significance in the MR analysis of IBD versus UC (Figure 5).

## 4 Discussion

In this paper, nine kinds of mitochondrial proteins causally related to IBD and its subtypes were unearthed by MR analysis. These results help to unearth potential targets for IBD therapy and also reveal the differences that exist at the mitochondrial level for subtypes of IBD. In the future, further mechanistic studies on the unearthed mitochondrial proteins are needed to reveal the pathways that induce IBD and why their proteins differ.

Hydroxymethylglutaryl‐CoA synthase (HMG‐CoA synthase) is a pivotal enzyme in the cholesterol biosynthesis pathway. Its primary function lies in catalyzing the conversion of acetaldehyde and acetyl‐coenzyme A into hydroxymethylglutaryl‐coenzyme A (HMG‐CoA), which serves as the initiator of cholesterol synthesis. The activity of HMG‐CoA synthase plays a crucial role in maintaining normal cholesterol levels and regulating lipid metabolism. Studies have demonstrated that rosuvastatin, an HMG‐CoA reductase inhibitor, exhibits the ability to mitigate the colitis response in mice induced by sodium dextran sulfate [34]. Furthermore, investigations have revealed a downregulation of HMG‐CoA synthase 2 (HMGCS2) expression in intestinal epithelial cells under the specific conditions of IBD relating to endoplasmic reticulum stress [35]. These findings align with the conclusions drawn in the present study.

NADH dehydrogenase [ubiquinone] flavoprotein 2 (NDUFV2) is encoded by the NDUFV2 gene expressed in human mitochondria. NADH dehydrogenase [ubiquinone] flavoprotein 2 is a subunit of the mitochondrial respiratory chain complex I, which is involved in the oxidative reduction of NADH to NAD+ and electron transmission to coenzyme Q in respiratory chain [36]. This process generates a proton gradient that is used to generate intracellular energy. However, its existence in association with IBD is reported for the first time, according to our limited search results so far.

Apoptosis‐inducing factor 1 is encoded by the AIFM1 gene and plays an important role in apoptosis [37]. A study indicated that AIFM1 is a core gene in ulcerative colitis‐associated colorectal cancer [38]. However, the specific mechanism of AIFM1's role in UC is unclear.

Pyruvate carboxylase is a mitochondrial enzyme concerned with pyruvate‐to‐pyruvate conversion as part of TCA cycle [39]. GrpE protein homolog 1 is a protein that is encoded by the HSPD1 gene. It plays an important function in the cell, mainly as a molecular chaperone protein, assisting other proteins to fold and assemble correctly. This helps maintain the stability and functionality of proteins in the cell. In addition, GrpE protein homolog 1 is also involved in the degradation and repair processes of intracellular proteins. tRNA pseudouridine synthase A is an enzyme, encoded by the TRUB1 gene. It plays an important cellular feature, primarily in the conversion of uridine to pseudouridine in tRNA molecules. Pseudouridine is a specialized nucleotide that can provide significant contributions to the construction and function of tRNAs [40]. tRNA pseudouridine synthase A activity contributes to the maintenance of the structural stability and biological function of tRNAs, which in turn affects protein synthesis and normal cellular function [41]. However, the mechanism of action of these proteins in UC needs to be further investigated.

MR research is less susceptible to confounding factors than previous observational research. And, it also avoids the large costs that need to be spent on adopting RCTs. The characteristics of this research could be summed up below: (1) The massive scale GWAS data sample ensures that the results of the study have a certain 1 reliability. (2) The methods of LD analysis, strongly correlated IVs, and outlier SNPs exclusion are effective in mitigating potential bias. (3) Both the multiple validity testing and the leave‐one‐out testing revealed relatively stable outcomes. The SNPs were symmetrically distributed in the funnel plot.

However, there are some limitations in this study: (1) The test of heterogeneity adopted in this paper is that both MR‐Egger regression and IVW method results are greater than 0.05. However, it is important to note that variations in analysis platforms, experimental conditions, population characteristics, and other IVs may also contribute to heterogeneity, which could not be avoided computationally in MR analysis. Therefore, the sources and causes of heterogeneity need to be investigated more fully and carefully in future studies. (2) An important limitation of our study is the predominant use of European‐ancestry GWAS data, which may limit the generalizability of our findings to other ethnic populations. Genetic architectures and allele frequencies can vary across populations, potentially leading to different causal estimates in non‐European groups. Future studies should aim to incorporate diverse population datasets as they become available to examine whether these mitochondrial‐IBD relationships hold across different ethnicities. (3) The number of IVs screened per exposure in this study was low (no more than 10). This is dictated by the sample size investigated by the mitochondrial protein‐related GWAS, and there is a need to expand the SNPs that can be used as IVs in the future by utilizing larger sample datasets for in‐depth analysis. (4) While our MR approach establishes causal associations, the specific biological mechanisms linking these mitochondrial proteins to IBD pathogenesis warrant further investigation. For example, the protective effect of NDUFV2 may relate to its role in maintaining mitochondrial respiratory chain function and reducing oxidative stress in intestinal epithelial cells. Similarly, the risk association with Peptide chain release factor 1‐like could reflect impaired mitochondrial protein synthesis affecting epithelial barrier integrity. These hypotheses could be tested in future experimental studies using intestinal organoid models or conditional knockout mice.

## 5 Conclusion

In this study, we analyzed GWAS dataset related to mitochondrial proteins and IBD using MR. Thirteen causal associations between mitochondrial proteins and IBD (including its subtypes) were revealed by MR analysis, and the results passed the tests of heterogeneity, pleiotropy, and directionality. Moving forward, investigating in greater depth the molecular processes involved in the heightened incidence of IBD related to these elements might contribute to the formulation of therapeutic ideas designed to reduce the risk of IBD incidence by improving mitochondrial function.

## Ethics Statement

The authors have nothing to report.

## Conflicts of Interest

The authors declare no conflicts of interest.

## Supporting information


**Table S1:** Summary of data source of 63 kinds of mitochondrial characteristics.
**Table S2:** suitable instrumental variables of 63 kinds of mitochondrial characteristics.
**Table S3:** The single nucleotide polymorphism selected for inflammatory bowel disease to perform Mendelian randomization analysis.
**Table S4:** The single nucleotide polymorphism selected for ulcerative colitis to perform Mendelian randomization analysis.
**Table S5:** The single nucleotide polymorphism selected for crohn's disease to perform Mendelian randomization analysis.
**Table S6:** Estimates of causal effect of inflammatory bowel disease on 63 kinds of mitochondrial characteristics.
**Table S7:** Estimates of causal effect of ulcerative colitis on 63 kinds of mitochondrial characteristics.
**Table S8:** Estimates of causal effect of crohn's disease on 63 kinds of mitochondrial characteristics.
**Table S9:** The results of MR‐PRESSO test of each Mendelian randomization study.
**Table S10:** Pleiotropy analysis of mendelian randomization analysis.
**Table S11:** Heterogeneity analysis of mendelian randomization analysis.
**Table S12:** Steiger test of mendelian randomization analysis.
**Table S13:** The single nucleotide polymorphism selected for inflammatory bowel disease to perform Mendelian randomization analysis (after screening).
**Table S14:** The single nucleotide polymorphism selected for ulcerative colitis to perform Mendelian randomization analysis (after screening).
**Table S15:** The single nucleotide polymorphism selected for crohn's disease to perform Mendelian randomization analysis (after screening).
**Table S16:** Estimates of causal effect of inflammatory bowel disease on mitochondrial characteristics after screening.
**Table S17:** Estimates of causal effect of ulcerative colitis on mitochondrial characteristics after screening.
**Table S18:** Estimates of causal effect of crohn's disease on mitochondrial characteristics after screening.
**Table S19:** Inverse‐variance weighted of inflammatory bowel disease and its subtypes on mitochondrial characteristics after screening (False Discovery rate).

## Data Availability

The data that support the findings of this study are available from the corresponding author upon reasonable request.
